# A Resource of Quantitative Functional Annotation for *Homo sapiens* Genes

**DOI:** 10.1534/g3.111.000828

**Published:** 2012-02-01

**Authors:** Murat Taşan, Harold J. Drabkin, John E. Beaver, Hon Nian Chua, Julie Dunham, Weidong Tian, Judith A. Blake, Frederick P. Roth

**Affiliations:** * Donnelly Centre for Cellular & Biomolecular Research, University of Toronto, Toronto, Ontario M5S-3E1, Canada; † Department of Biological Chemistry and Molecular Pharmacology, Harvard Medical School, Boston, Massachusetts 02115; ‡ Mouse Genome Informatics, The Jackson Laboratory, Bar Harbor, Maine 04609; § Institute of Biostatistics, School of Life Sciences, Fudan University, Shanghai 200433, P. R. China; ** Center for Cancer Systems Biology, Dana Farber Cancer Institute, Boston, Massachusetts 02115; †† Samuel Lunenfeld Research Institute, Mt. Sinai Hospital, Toronto, Ontario M5G-1X5, Canada

**Keywords:** function prediction, gene function human, Gene Ontology, machine learning

## Abstract

The body of human genomic and proteomic evidence continues to grow at ever-increasing rates, while annotation efforts struggle to keep pace. A surprisingly small fraction of human genes have clear, documented associations with specific functions, and new functions continue to be found for characterized genes. Here we assembled an integrated collection of diverse genomic and proteomic data for 21,341 human genes and make quantitative associations of each to 4333 Gene Ontology terms. We combined guilt-by-profiling and guilt-by-association approaches to exploit features unique to the data types. Performance was evaluated by cross-validation, prospective validation, and by manual evaluation with the biological literature. Functional-linkage networks were also constructed, and their utility was demonstrated by identifying candidate genes related to a glioma FLN using a seed network from genome-wide association studies. Our annotations are presented—alongside existing validated annotations—in a publicly accessible and searchable web interface.

An abundance of high-throughput laboratory techniques and computational methods has led to a deluge of human genomic and proteomic data (Bieri *et al.* 2007; Crosby *et al.* 2007; Eppig *et al.* 2007; Nash *et al.* 2007). Even when an individual researcher can assemble all available evidence, they are left with the task of weighing this evidence to infer likely functions for genes of interest.

Fewer than one-third of human genes have a Gene Ontology (GO) annotation based on evidence derived from specific study of that gene (as opposed to prediction; see Figure 2), providing little guidance to researchers wanting to investigate sparsely annotated genes. Computational integration of diverse evidence can help in assigning function, and ideally these inferences can reflect the shades of gray in our current knowledge, as opposed to the “black or white” annotation that is most appropriate for archival annotation.

Automated annotation has traditionally relied on sequence similarity to transfer function from one gene to another, but homology-based approaches fail in the common scenario in which a gene/function relationship is unknown or poorly characterized in all detectable homologs. Toward complementary approaches, integration of diverse data types have been adopted for use in making predictions of gene function (Troyanskaya *et al.* 2003; Deng *et al.* 2004; Joshi *et al.* 2004; Karaoz *et al.* 2004; Lanckriet *et al.* 2004; Wong *et al.* 2004; Huttenhower *et al.* 2009; Linghu *et al.* 2009; Bologna *et al.* 2011) and modeling term-to-term relationships [(King *et al.* 2003; Sokolov and Ben-Hur 2010); see refs. (Murali *et al.* 2006) and (Huttenhower and Hofmann 2010) for general reviews on the concepts]. For example, one large effort (‘MouseFunc’) was undertaken by a consortium of research groups to compare function prediction methods using standardized training and testing datasets for *Mus musculus* (Pena-Castillo *et al.* 2008).

Here we integrated an extensive set of *Homo sapiens* data and inferred quantitative associations between 21,341 human genes (12,925 of which had no existing associations on the basis of direct–ie, nonpredicted–evidence) and each of 4333 GO terms. Our models exploit both gene features and gene−gene relationships by using both guilt-by-profiling (GBP) and guilt-by-association (GBA) approaches to function prediction (Taşan *et al.* 2008; Tian *et al.* 2008). We provide estimates of our models’ accuracy, including a prospective evaluation in which we consider annotations that were made after our training data set was assembled. Literature-based follow-up investigations are performed for a sample of high-confidence novel predictions. In the course of making these predictions, we constructed multiple functional linkage networks [FLNs–where an edge between two genes indicates some level of shared function (Lee *et al.* 2004)], capturing different categories of biological relationships. We find that FLNs are independently useful, which we illustrate here by identifying candidate glioma-related genes given only “seed” glioma-related genes identified from systematic unbiased genome-wide association (GWA) studies. All gene/term prediction scores from this project—as well as our FLNs—are made freely available to the public via a web-accessible resource (Beaver *et al.* 2010), which has been adapted here to host quantitative function annotations for *H. sapiens*.

## Materials and Methods

### Genomic data integration

#### Genes:

We used 36,396 Ensembl Gene IDs as the initial set of identifiers for *H. sapiens* genes, which was further reduced to 21,341 genes as described in the section *Predictive features*. All mappings between alternative identifiers and Ensembl Gene IDs were performed using the Synergizer translation resource (Berriz and Roth 2008).

#### Predictive features:

We downloaded 6121 protein domain signatures from Interpro (Mulder *et al.* 2005) that could be associated with any one of the downloaded Ensembl Gene IDs. We also identified 2542 disease descriptions from Online Mendelian Inheritance in Man [OMIM (Hamosh *et al.* 2005)] that were assigned to Ensembl Gene IDs. Phenotype terms from the Mammalian Phenotype Ontology (Smith *et al.* 2005) were downloaded and transferred from *M. musculus* to human via a homology mapping as provided by Mouse Genome Informatics (Eppig *et al.* 2007); because many of the phenotype terms are quite specific and have a nearly one-to-one mapping to GO BP terms, we limited our use of phenotype terms to only those at the top level of the phenotype ontology: 34 such terms mapped to our Ensembl Gene IDs. Phylogenetic profiles were built for each gene on the basis of ortholog presence in the genome of any of 38 possible organisms, as determined by the default threshold used by InParanoid (O’Brien *et al.* 2005). The 21,341 genes used in this study had at least one “gene-centric” feature listed previously.

Correlation coefficients were computed for pairs of genes using all Affymetrix HG-U133A (PL96) GEO (Barrett *et al.* 2009) datasets for which CEL files were available (97 datasets in total). Each dataset was preprocessed separately using GCRMA background correction, normalization, and probeset summarization (Wu *et al.* 2004), with probeset-to-gene mappings provided by BrainArray Custom CDFs (Version 11) (Dai *et al.* 2005). Minimum-jackknife-1 Pearson correlation coefficients were computed for each gene-pair in each of the 97 datasets. The 35,948 protein interactions downloaded from HPRD (Keshava Prasad *et al.* 2009) were mapped to Ensembl Gene IDs; filtering of this dataset to remove homo-dimers left 33,811 protein-protein interactions within our gene set.

#### GO terms and associations:

Our models were trained using the human GO associations [from the GOA project (Barrell *et al.* 2009)] from August 2008, with associations from May 2009 used for the prospective evaluation. All IEA associations were removed, and the remaining associations were up-propagated through the GO graph. Only those 4333 GO terms with at least 3 and at most 300 distinct (non-IEA) gene associations were included for this study. We used the 8416 genes containing some non-IEA association to any one of these terms as the training set of genes, with the remaining 12925 genes being the undetermined set. Terms were divided into 12 categories, as described previously.

### Model construction and evaluation

#### GBP models:

In total, we used 8735 gene-centric predictive features: 6121 distinct InterPro IDs, 2542 OMIM Morbid Map IDs, 34 Mammalian Phenotype Ontology IDs, and 38 species used to build phylogenetic profiles. Collectively, we call these the GBP feature set.

For each GO term, we trained a separate random forest classifier as an ensemble of 100 recursive partitioning or decision tree classifiers, using the GBP feature set and the training set of genes as gold-standard positive examples. The classifiers produced scores for each gene/term combination: predictive scores for undetermined genes, and out-of-bag (OOB) scores^2^ for training genes. Precision-vs-recall performance using OOB scores was calculated for each GO term, then combined by term category to obtain aggregate measures of performance over all GO terms (supporting information, Figure S1).

#### GBA models:

Twelve GBA classifiers were built (one for each GO term category), with each producing a separate FLN that provides scores to all pairs of genes (*i.e.* $\left(\begin{array}{c} 21341\\ 2 \end{array}\right)=227708470$ gene-pair scores). Here again we used random forests, but in this case the set of objects to be scored consisted of gene pairs (rather than genes).

Among the 8416 training-set genes, gene pairs that shared any GO term within the same category were used as positive training examples of functionally-linked genes. To illustrate by example, our BP [31,100] FLN was constructed using gene pairs that shared any one (or more) associations to any BP term with fewer than 101 (non-IEA) total associations. Such pairs were deemed functionally linked and given a score of “1” in the training network. All remaining pairs among the 8416 training genes were considered *not* to be functionally linked (with respect to the current GO term category) and were scored as “0” in the training network. Predictions of edge weights for all other pairs of genes ($\left(\begin{array}{c} 21341\\ 2 \end{array}\right)-\left(\begin{array}{c} 8416\\ 2 \end{array}\right)=192298150$ pairs) are then made using random forest models trained on these reference networks, and the $\left(\begin{array}{c} 8416\\ 2 \end{array}\right)$ OOB edge scores for the training edges are used for assessing model performance (described below).

For each of our Interpro, OMIM, Mammalian Phenotype Ontology, and phylogenetic tree datasets, shared patterns of annotation between pairs of genes were scored using a method that is functionally similar to the PhenoBlast score used previously for measuring similarity between pairs of *Caenorhabditis elegans* strains (Gunsalus *et al.* 2004). The score between two genes is computed as $\sum^{\text{​}}f_{i}^{2}$, where *f_i_* is the frequency of the *i*-th feature shared by both genes. Each such score was then discretized by placing gene-pairs into bins corresponding to the membership in the top 10%, 20%, 30%, 40%, and 50% quantiles. Thus, each of these datasets led to five binary features used in training our functional-linkage models.

Already in binary form, protein−protein interactions from Human Protein Reference Database (HPRD) are included as a predictive feature. Each expression dataset was also discretized by using the top 1%, 5%, and 10% quantiles, leading to three binary features per expression dataset.

To make individual gene/term predictions from our FLNs, we use a probabilistic approach. For GO terms in evaluation categories with association counts in the ranges [11, 30], [31, 100], and [101, 300], we compute gene/term scores one term at a time, starting by identifying the “core set” of genes that have positive associations with the current GO term. A probability density function is constructed from the edge weights between all pairs of genes in the core set (forming a clique), using a Gaussian kernel density estimation (the “core-to-core” distribution). A “pseudo-count-like” edge weight uniformly distributed over [0,1] is added to this collection to prevent the density estimators from dropping to zero during numerical computations with a reasonable kernel bandwidth. Similarly, the weights between all pairs of noncore training genes and the core set of genes are used to estimate a “noncore-to-core” distribution. Finally, a gene not in the training set is scored (for the current GO term) by identifying all edges between that gene and core set genes; the log-likelihood ratio (LLR) of these edges having arisen from the “core-to-core” relative to the “noncore-to-core” distributions is then computed using the estimated probability density functions.

For GO terms with 10 or fewer gene associations, the process is similar to that described, except that “core-to-core” and “noncore-to-core” distributions are estimated from core-to-core and noncore-to-core gene-pair sets pooled across all GO terms in the evaluation category. We selected this method after empirical observation that the limited number of core genes for many such GO terms led to poor density function estimation (results not shown).

Performance of annotation transfer by LLRs was estimated using a leave-one-out (LOO) approach: each gene in the training set was scored using the OOB edge weights computed during random forest construction, and distributions were re-estimated after excluding all edges incident to the current held-out gene. The transferred LOO scores for each GO term were then evaluated similarly to the scores produced by the GBP models (Figure S2).

#### Combined models:

We combined the GBA and GBP scores for each gene/term via logistic regression, referring to the combined scores as “HF” scores. The general form of the combination is:

$$LO_{i}^{HF}=\alpha \cdot LLR_{i}^{GBP}+\left(1-\alpha \right)\cdot LLR_{i}^{GBA}+LO^{prior},$$

where $LO_{i}^{HF}$ is the posterior HF log-odds and $LLR_{i}^{GBP}$and $LLR_{i}^{GBA}$ are the log-likelihood ratios of the GBP and GBA models, all for each gene *i*. *LO^prior^* is the prior log-odds of a gene being associated with a particular term (invariant across genes for each term).

The α-term in the aforementioned equation is systematically optimized to maximize the LOO estimates of the area under the PR curve (average precision) for each term. For GO terms in evaluation categories with association counts in the ranges [11,30], [31,100], and [101,300], the α coefficients are determined separately for each GO term. To avoid overfitting the coefficients for the GO terms in evaluation categories in the range [3,10], a single coefficient is found for all GO terms within the category (*i.e.* α_BP[3,10],_ α_CC[3,10],_ α_MF[3,10],_).

### Prospective evaluation

For prospective evaluation, each term was evaluated individually (as mentioned previously), but with changes to the set of genes used for the evaluation. First, all existing positive associations made prior to the start date were removed from each term’s list of gene scores. Second, only those genes that received *any* association (*i.e.*, to any other term in the GO) in the time since the start date were retained; there were 1170 such genes. The idea here is that any new association for a gene made in the prospective time window indicates that this gene had some curation done and thus had the opportunity to be newly associated with at least one term (and possibly more). Genes not receiving any new association in that time window were not considered for prospective validation. Aggregate prospective performance across all GO terms is shown in Figure 6.

### Selection of 36 predictions for literature follow-up

Three gene/term pairs from each of the term categories were chosen for a literature-based follow-up. We considered only gene/term pairs for which the gene had no known or predicted (*i.e.*, IEA-based) function within the same GO branch—we call these “novel” predictions. The top three HF scores within each term category after this filtering were then chosen. A list of these 36 predictions, along with their assessments and literature references, is available as Supplementary Information.

### Microarray differential expression analysis

When evaluating differential expression for our glioma network follow-up, we used Welch’s two sample *t*-tests, followed by a multiple-testing correction (Benjamini and Hochberg 1995). We note that the Bredel *et al.* (2005) dataset was not included as part of our microarray co-expression data set used for training our FLNs (to address circularity in the discovery process).

### Computational tools

Random forest code was written in Java for this study, and all statistical analyses (*e.g.*, model performance estimates) were performed using R. All software used in generating this resource is freely available upon request.

## Results

### Data integration

**Figure 1:**
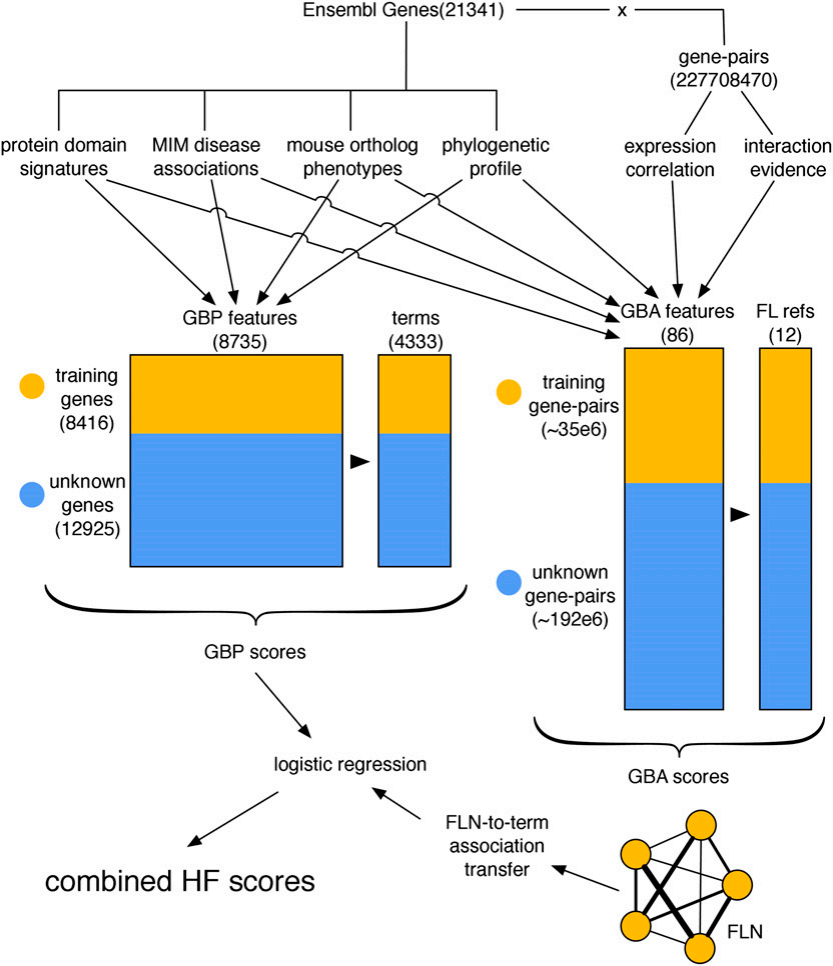
General strategy for the combined GBP and GBA approach. (GBP: guilt-by-profiling, GBA: guilt-by-association, HF: human function, FLN: functional-linkage network.)

All data collected were from public sources and fell into the two broad classes of gene property and gene-pair relationship (Figure 1, Table 1, Table 2), and 4333 GO terms were chosen for classification (see *Materials and Methods* for a description of the selection criteria). Terms were separated into 12 distinct term categories on the basis of the aspect of function it describes, that is, biological process (BP), cellular component, or molecular function (MF), and its breadth, that is, the number of unique genes to which it has been assigned ([3,10], [11,30], [31,100], or [101,300] non-IEA associations, following the paradigm introduced in Pena-Castillo *et al.* 2008, Table 3). These distinctions allow us to separately study inferences to GO terms of similar “type” and permit an understanding of how performance differs among these types. Gene features and GO term associations were gathered in 2008, with the prospective evaluation reported here based on term associations made approximately 1 year later (2009). All associations with IEA evidence codes were removed from this study (forcing our models to rely on ground truth rather than on previous predictions), with the remaining association types used for model training being distributed as shown in Figure 3.

**Figure 2:**
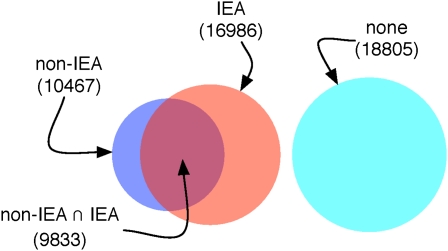
Distinct Ensembl Gene IDs grouped into broad classes depending on status of association with at least one GO term.

**Table 1:** Number of distinct genes (21,341 total genes) with at least one “gene-centric” feature attributed to it for each predictive data type

Data Type	No. Genes	No. Features
Domain signature pattern	15,274	5063
MIM disease association	1765	2125
Phylogenetic profile	20,964	38
Phenotype association	5162	34

MIM, Mendelian Inheritance in Man.

**Table 2:** Number of distinct genes (of 21,341 total genes) appearing in at least one “gene-pair-centric” data type

Data Type	No. Genes
Expression profiles	11911
Interaction evidence	7725

Details of how each such data type is split into predictive features are given in *Materials and Methods*.

**Table 3:** Number of terms in each of the 12 evaluation categories

Num Assoc	BP	CC	MF
[3,10]	1490	234	686
[11, 30]	694	121	231
[31, 100]	374	85	158
[101, 300]	177	34	49

BP, biological function; CC, cellular component; MF, molecular function.

**Figure 3:**
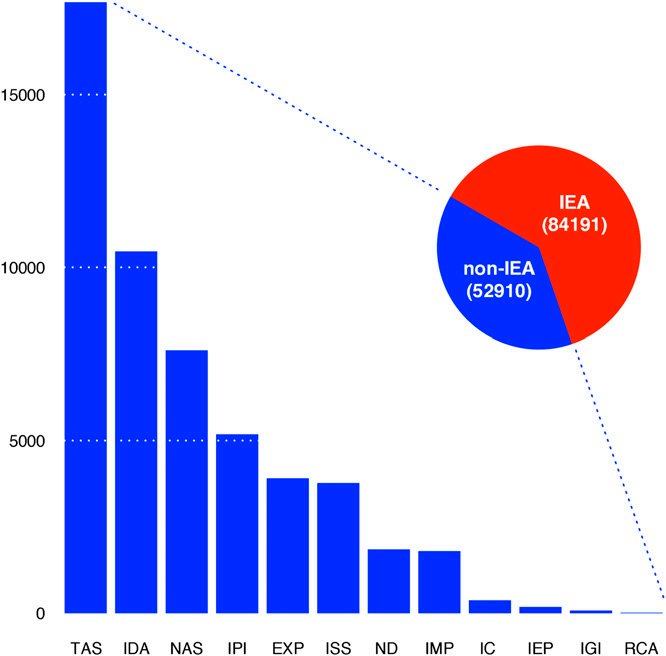
Counts of association types between Ensembl Gene IDs and GO terms (by GO evidence code). The barplot represents the evidence code distribution within the smaller (non-IEA) slice of the pie.

### Quantitative association of 21,341 *H. sapiens* genes to 4333 GO terms

Predictions of gene/term associations were made by combining GBP and GBA approaches. Quantitative scores describing the strength of association between each GO term and human gene were computed (for a total of 4333⋅21,341 ~ 92 × 10^6^ gene/term scores).

In the GBP approach (relying on “gene-centric” data), a separate random forest classifier (Breiman 2001) was constructed for each GO term, providing direct gene/term predictions that we call the “GBP set.” The GBA approach is complementary to GBP in that it focuses on “transferring” annotation from one gene to another via evidence of a biological relationship between the genes. To this end, data best represented as “edge-centric” (*e.g.*, protein−protein interactions) were used to construct FLNs, graphs describing relationships (edges) between genes (vertices), where edge weights reflect confidence levels of two genes sharing a function (Lee *et al.* 2004). We constructed 12 probabilistic FLN networks (one for each of the aforementioned 12 categories), also using random forests (one per FLN). Associations between each gene *g* and each term *t* were then transferred probabilistically by examining the strength of connection between *g* and a ‘core set’ of genes already annotated with term *t* (details in *Materials and Methods*). The collection of gene/term scores derived from this process is called the “GBA set.”

Finally, the GBP and GBA scores were combined for each GO term via a logistic regression model optimized to maximize precision *vs.* recall (PR) performance (see *Materials and Methods*), resulting in the human function (HF) set of quantitative confidence scores.

### Predictive performance

**Figure 4:**
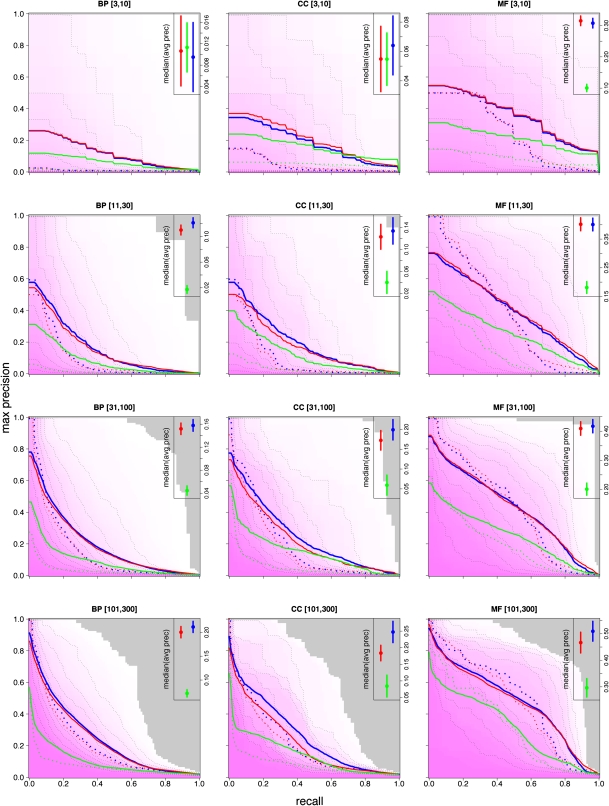
Aggregated HF performance for each of the 12 GO term categories. Contour lines indicate the fraction of GO terms that met or exceeded each cumulative precision *vs.* recall performance point. Contours are shown for median GO term (50% contour; heavy blue dashed line) and every other decile of GO terms. The mean performance (heavy blue solid line is also shown. Gray area exceeds performance of all classifiers. Red and green lines indicate median and mean performances (dashed and solid, respectively) for GBP and GBA classifiers. The median of average cumulative precision across all classifiers in each category is shown in inset (error bars show standard error of the median).

**Figure 5:**
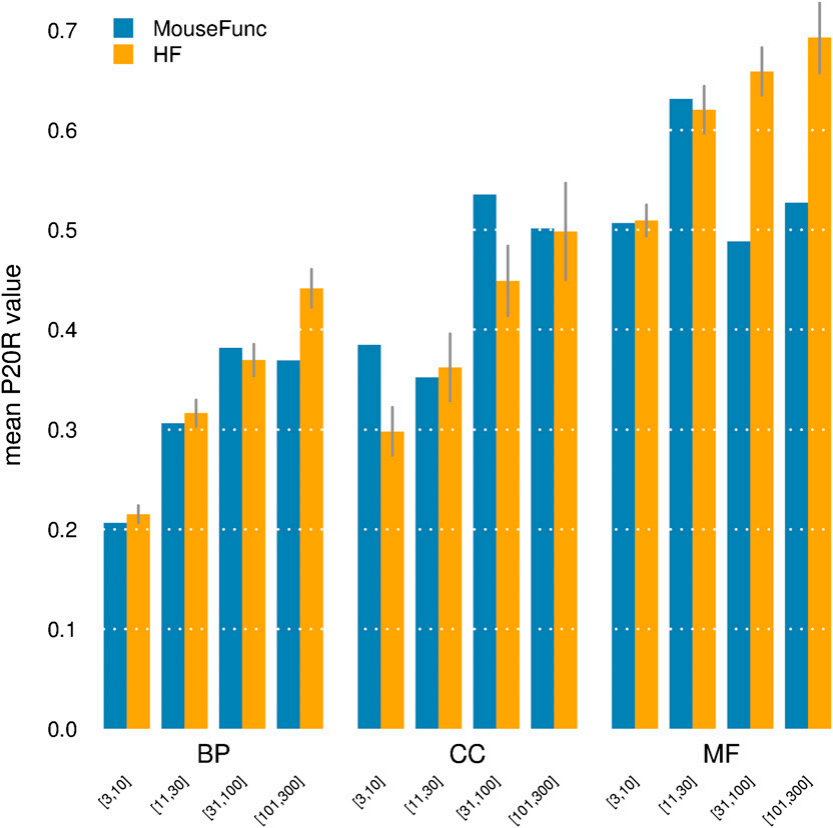
Comparing current performance with previous mouse gene function predictions. Precision at 20% recall for best-performing groups of the MouseFunc project (Pena-Castillo *et al.* 2008) was compared with HF scores in this study. Differences reflect a combination of both algorithms and datasets used but indicate that overall prediction performance is at least comparable and for some term categories represents a substantial improvement. Error bars indicate standard error of the mean; variance across terms for MouseFunc not available, preventing error bar computation.

Our predictions were evaluated (using a cross-validation scheme) for each term, and for all three sets of scores (GBP, GBA, and HF). To obtain a global view of performance, evaluation measures were aggregated into the 12 term categories and illustrated using contour analysis that shows the characteristic trade-off of accuracy *vs.* recovery for predictive models (Figure 4, Figure S1, and Figure S2). For a given score threshold τ, *precision* is the proportion of (known) “true” gene/term pairs among all gene/term pairs scoring above τ; *recall* is the proportion of “true” gene/term pairs scoring above τ among all possible “true” gene/term pairs. The combination of GBP and GBA scoring methods provide an increase in average precision for many different term types (insets of Figure 4). We also find that our performance (in terms of precision) compares favorably with the best performance achieved from the MouseFunc study (Figure 5), with mean precision at 20% recall (P20R) ranging between approximately 20% and 70%.

**Figure 6:**
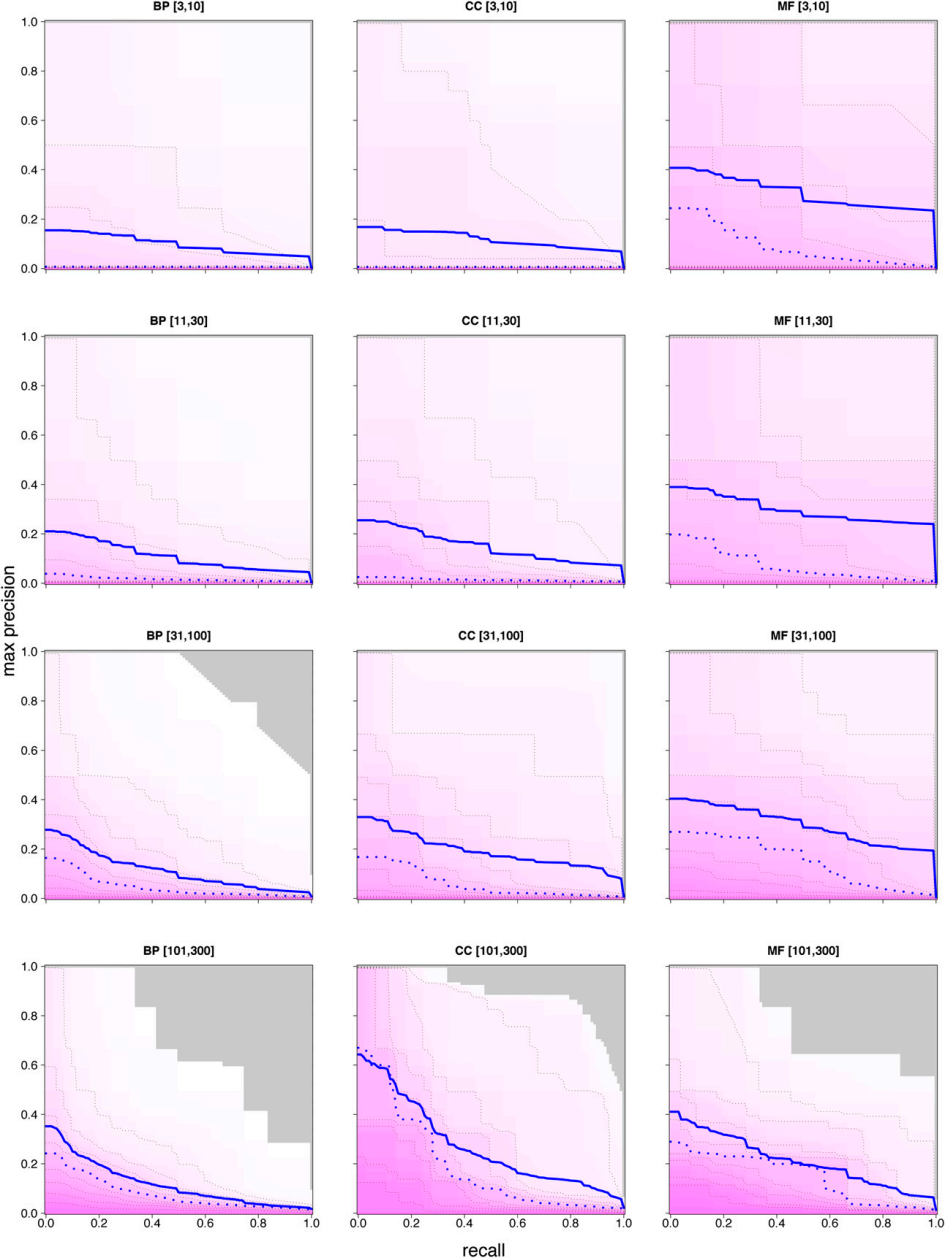
Prospective evaluation of precision *vs.* recall. Predictions made on July 19, 2008, evaluated on new associations made between July 19, 2008, and May 28, 2009. Dashed lines indicate each 10% contour, heavy dashed line is median (50% contour), and the heavy blue solid line is mean performance. Contours indicate what fraction of classifiers in the evaluation category exceeded the shown performance. Gray area exceeds performance of all classifiers.

Our HF predictions were also evaluated *prospectively* using gene/term associations made after the start date. Because all genes will not have been studied in this time window (and thus not given the opportunity to gain new annotations), we evaluated performance for the subset of 1170 genes that gained at least one new GO annotation during this period (Figure 6). Recall for each term was calculated using only gene/term pairs recently (*i.e.*, in the time window) assigned to that term. For lower recall levels (*e.g.*, <20%), our precision generally ranged between 20% and 40%.

### Literature-based validation of top novel predictions

Having evaluated the performance of quantitative annotations by cross-validation and prospective analysis, we wished to more carefully examine the most interesting HF predictions, that is, those that are both novel and most likely to be correct. To restrict ourselves to the most novel predictions, we excluded any predictions in which the gene had previously been annotated to any GO term in the same branch as the predicted GO term. We further excluded gene/term pairs that had previously been predicted (on the basis of annotation with an IEA evidence code). For each of the 12 GO categories, we examined literature related to the three top-scoring novel predictions, leading to 36 high-scoring gene/term predictions from the HF model for in-depth literature evaluation.

Of the predictions examined, we deemed 14 to be “true” or “likely true” (9 and 5, respectively) given evidence available in the current body of published literature. The success rate corresponds to 14 of 36 (39%) and thus appears similar to our prospective estimates of precision (described previously). Of the remaining predictions, 14 were unclear, in that we found no existing literature that either supported or refuted the prediction. These predictions can be viewed as hypotheses for further study. The final 8 predictions were either “likely false” (6) or “false” (2). (A full list of the 36 literature-evaluated predictions and chosen categories, with PubMed IDs, where available, is provided in File S1 for this article.)

### FLNs for functional gene set expansion

In addition to their contribution toward predicted gene/term associations, FLNs have previously been shown to be valuable tools for elucidating relationships between genes [*e.g.*, predicting genetic interactions via patterns of functional linkage (Lee *et al.* 2010)]. We further find that FLNs are useful for identifying disease-associated candidates by extracting subsets of genes that were functionally linked with particular seed genes of interest.

As an example of how “induced subgraphs” from FLNs can aid in annotation of genes even in the absence of any particular seed set of genes, we selected the single gene *POLR2J2*, which was found to be “likely true” in the aforementioned literature evaluation for association with the GO MF term “DNA-directed RNA polymerase complex” (GO:0000428). Considering only the edges scoring in the top 0.1% of the FLN for the term category BP [3,30], the 3 linked neighbors of *POLR2J2* are *POLR2J3*, *POLR2J*, and *POLR1C* (Figure S3). The nonseed genes with prefix *POLR2J* are each subunits of the RNA Pol II complex, and *POLR1C* is a subunit of the RNA Pol I complex. We note that *POLR2J2* has no cataloged interaction partners in the HPRD (Keshava Prasad *et al.* 2009), limiting the prospects of GBA via physical or genetic interactions alone. Although this is a case of an already named and characterized gene, it serves to illustrate the (1) automated identification of strong functional hypotheses and (2) identification of groups of additional genes enriched for functional relationships with a particular incompletely annotated gene of interest.

**Figure 7:**
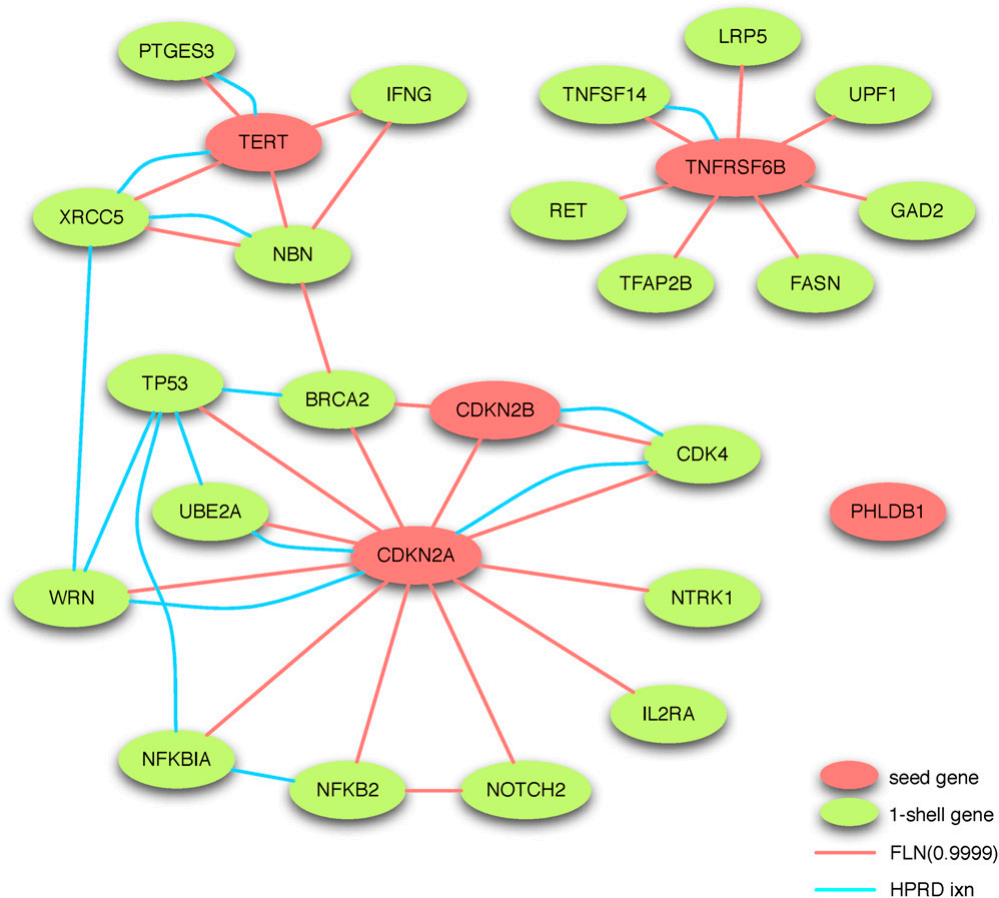
Illustrated use of the human FLN to identify additional candidate genes from a set of seed genes. This network was identified using five glioma genes identified in unbiased genome-wide association studies (in red). The FLN used was the top 0.01% of edges (in red) from the BP [3,30] FLN. To illustrate that the FLN extends beyond known protein interactions, all cataloged HPRD interactions between these 26 genes are shown as blue edges.

To illustrate how one may use FLNs to identify novel candidate disease genes [an idea supported by recent similar work in ref. (Lee *et al.* 2011)], we examined a seed set of candidate genes identified from a meta-analysis of GWA studies examining glioma susceptibility (Shete *et al.* 2009). Five of the six genes identified by the meta-analysis were among the 21,341 genes examined in our work, which we used as seeds in the BP [3,30] FLN with an edge-weight threshold retaining only the top 0.01% of edges. The resulting subgraph induced by this seed set of five glioma-associated genes recruited 21 additional genes (Figure 7). Genes common to many forms of cancer were among those found (*e.g.*, *BRCA2*, *TP53*, *CDK4*, *XRCC4*, none of which were seeds originating from the GWAS meta-analysis). Two seed-centered components (one from the seed *TERT*, the other from the seeds *CDKN2A* and *CDKN2B*) are connected via a functional link between the two nonseed genes *BRCA2* and *NBN*. This approach generalizes the strategy of inducing disease networks from known disease-associated seed genes using physical interaction data (Pujana *et al.* 2007).

To illustrate the greater potential for detection of related genes when FLNs are used, as opposed to using only protein interactions alone, we also display (Figure 7) all interactions cataloged in HPRD between the 26 genes in our glioma network. Among interesting candidate genes retrieved using the FLN, the *NBN* gene had previously been postulated to have a role in nonglioma forms of cancer, and recent evidence has (independently from the GWA studies) implicated *NBN* variants in pediatric glioblastoma (Piekutowska-Abramczuk *et al.* 2009). Also of interest is the recovery of the *LRP5* gene, a low-density lipoprotein receptor-related protein that has been identified as potentially oncogenic (Li and Bu 2005) and part of the cancer-drug-targeted Wnt/beta-catenin signaling pathway (Luu *et al.* 2004). We note that both the *NBN* and *LRP5* genes would not have been recovered using the HPRD interaction network alone (with a “1-nearest-neighbor” expansion). To further analyze the value of our glioma network, we examined a microarray expression dataset from an experiment comparing (among other tumor types) glioblastoma samples and normal brain tissue [(Bredel *et al.* 2005) NCBI GEO (Barrett *et al.* 2009) record GDS1813]. Of the 21 genes in our glioma network for which we could compute test statistics, we found that 10 show differential expression (FDR < 0.1), including the *NBN* and *LRP5* genes, shown in Figure S4.

### Resource availability

Our predictions are made available as a resource to the community via an online web-accessible and searchable browser (Beaver *et al.* 2010), available via http://func.mshri.on.ca/human, with periodic data updates (trained on current gene annotations). Our FLNs, not included in the File S1 because of file size constraints, are available for download at http://llama.mshri.on.ca/~mtasan/HumanFunc_publication_support.

## Discussion

### Combining GBP and GBA methods allows full data exploitation

Our quantitative gene function annotations combine both gene-centric and gene-pair relationship evidence. The contribution of relationship-based (GBA) scoring tends to increase as the terms become more specific. Given that gene-centric (GBP) models are trained separately for each GO term, it stands to reason that performance must degrade for GO terms with very few annotated genes to use as positive training examples. By contrast, FLNs are trained by aggregating information from many related GO terms. This lends support to the notion of using the FLNs for inferring specific functions. Also we find that terms in the MF (molecular function) branch of the GO vocabulary often rely more heavily on the GBP scores; this is likely to be due, at least in part, to the strong association between protein domain pattern signatures and specific biochemical functions.

### Emphasis on high precision at low recall

We expect that a common use of this resource will be experimental biologists examining only the top few quantitative annotations for any gene or term to generate hypotheses for testing. Presentation of a ranked list of annotations, each in the context of underlying evidence, permits filtering based on (often extensive) user knowledge that was not modeled by our approach. Thus, our GBP and GBA predictions are combined to maximize average precision, which is largely driven by the accuracy of the top predictions.

#### Cross-validation vs. prospective performance evaluation:

Differences between the training performance estimates and the prospective estimates reflect both the bias in the state of the associations at the start date (*i.e.*, some genes and terms are better-studied than others) and the types of genes and terms recently curated by GOA. Even limiting the evaluated genes to those that have gained some new annotation in the time period, there are several reasons why one might expect to see lower precision-recall performance for the prospective evaluation as compared to that seen with cross-validation. First, each freshly-annotated gene gained an average of only 3.1 new GO term associations, which is lower than the total number of terms that we might ultimately expect to be added for each gene. Second, recently-annotated functions may be intrinsically harder to predict (*i.e.*, where the function is obvious, annotations will tend to occur at an earlier time). Third, recent advances may be on functions that were less well-studied previously, so that the training data are sparsely available or is not representative.

### Variable importance

As part of our GBP process, we obtain a variable importance measure for each predictive feature, measuring that feature’s contribution to the performance of the classifier. These can assist in understanding *why* a prediction has been made, possibly suggesting a validation experiment or simply providing more general clues to help guide the researcher. Strong evidence of particular features leading to accurate gene/term associations may lead to the establishment of empirically derived annotation-transfer rules. As an example, the gene *RAET1L* was predicted to have MHC class I receptor activity, and based solely on the available literature, this prediction was deemed “unclear.” That two of the top three Interpro protein domain pattern signatures with high importance for predicting MHC class I receptor activity had been assigned to this gene (a MHC class I alpha chain pattern (IPR001039) and a MHC classes I and II-like antigen recognition pattern (IPR011162)) yields insight into why this prediction was made, and can guide curators in their decision on whether to make a GO term association for this gene. Such feature identification can also be used to help streamline future prediction efforts [by focusing efforts on those predictive features that are most informative for the task at hand (Ko and Lee 2009)].

### A novel method for annotation transfer through FLNs

To derive GBA scores for each term, we use the edge weights of the appropriate FLN to transfer associations from genes currently associated with that term (the “core set”) to those genes with “unknown” association status. This process calculates, for each candidate gene in turn, a likelihood ratio that measures whether the distribution of weights connecting the candidate to the core set is more consistent with ‘core-to-core’ pairs (that are known to share the function) than ‘core-to-non-core’ pairs (that are not known to share the function; see *Material and Methods* for details). We have previously experimented with other forms (see Wang and Marcotte 2010 for a review) of association transfer from FLNs, specifically, using average weight of (the top-*n*) edges linking “unknown” genes to “core set” genes (Tian *et al.* 2008; Taşan *et al.* 2008) and have found the likelihood-ratio approach applied here to be more robust and empirically better-performing than the ‘top-*n*’ approach in cross-validation evaluation (results not shown).

### Utility of FLNs

Physical interaction networks have been shown to help transfer gene function (Schwikowski *et al.* 2000; Letovsky and Kasif 2003; Gunsalus *et al.* 2005), and extending these approaches to include predicted *functional* interactions can drastically increase the power of discovery. The STRING database (Szklarczyk *et al.* 2011) is an example of a publicly accessible repository of predictions of functional interactions made through various evidence sources (*e.g.*, literature co-citation or existing protein−protein interaction reports). Our functional linkage networks differ from those in STRING in that we make predictions for all pairs of genes at different levels of resolution and with respect to different types of functional links (*e.g.*, BP—a shared role in some biological process *vs.* MF—sharing a particular molecular activity). When comparing the STRING (v9.0) interaction scores with those of each of our 12 FLNs, we see a significant correlation in each case (*P* < 3 × 10^−12^ for all 12 correlations measured). However, these correlations were relatively weak, with Pearson correlations ranging from *r* = 0.07 (category cellular component [3,10]) to *r* = 0.20 (category MF [3,300]). Although STRING provides highly reliable functional associations between genes, it is limited to only those pairs with strong scores (~3.3 million in STRING v9.0, as opposed to the ~200 million in each of the 12 FLNs described previously). A larger range of scores can provide additional information, for instance, establishing densities of scores for use in label propagation (as described in the section *A novel method for annotation transfer through FLNs*), thus we see a continued role for our FLNs in ongoing research.

Biological networks can reveal previously unknown players in the etiology of a phenotype, solely based on of their proximity in a FLN to known genes associated with the phenotype. For example, a seed set of genes associated with breast cancer has been successfully used to identify additional disease genes via protein interaction relationships (Pujana *et al.* 2007). Here we extend this notion in two ways: First, we use edges that aggregate multiple sources of evidence about pairwise functional relationships. Second, our predictive models include *essentially all* genes, including those that have neither existing physical interaction evidence nor existing GO annotations.

## Supplementary Material

Supporting Information
