# Supplementary material for: A Resource of Quantitative Functional Annotation for Homo sapiens Genes
Source: G3 (Bethesda). 2012 Feb 1;2(2):223–33. doi: 10.1534/g3.111.000828 (PMC3284330; doi:10.1534/g3.111.000828)
Supplement: Supporting Information [file supp_2_2_223__index.html]

Supporting Information 

# A Resource of Quantitative Functional Annotation for *Homo sapiens* Genes

## Supporting Information for Tasan *et al.*, 2012

**Files in this Data Supplement:**

- Supporting Information - Figures S1-S4 (PDF, 2.9 MB)
- Figure S1 - Aggregated GBP performance for each of the twelve GO term categories (PDF, 790 KB)
- Figure S2 - Aggregated GBA performance for each of the twelve GO term categories (PDF, 623 KB)
- Figure S3 - Network identified using 1 seed gene (in red) and the top 0.1% of edges from the BP 3, 30 FLN (PDF, 52 KB)
- Figure S4 - 26 glioma network genes' mean microarray values in normal brain and glioblastomas (PDF, 790 KB)
