# Supplementary material for: A Resource of Quantitative Functional Annotation for Homo sapiens Genes
Source: G3 (Bethesda). 2012 Feb 1;2(2):223–33. doi: 10.1534/g3.111.000828 (PMC3284330; doi:10.1534/g3.111.000828)
Supplement: Supporting Information [file supp_2.2.223_000828SI.pdf]

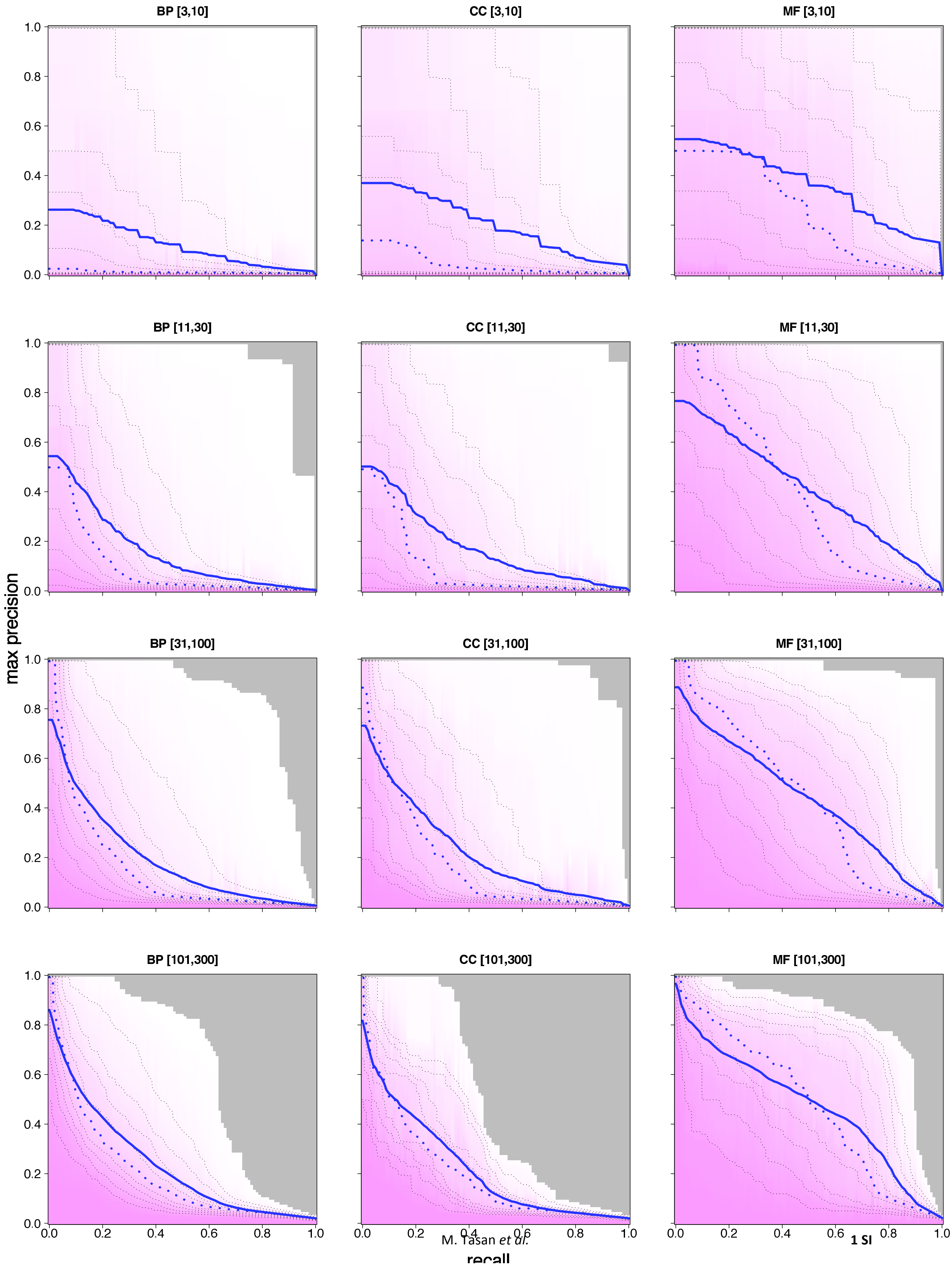
BP [3,10]
CC [3,10]
MF [3,10]
BP [11,30]
CC [11,30]
MF [11,30]
BP [31,100]
CC [31,100]
MF [31,100]
BP [101,300]
CC [101,300]
MF [101,300]
max precision
recall
0.0
0.2
0.4
0.6
0.8
1.0

Figure S1 Aggregated GBP performance for each of the twelve GO term categories. Dashed lines indicate each 10% contour, heavy dashed line is median (50%), heavy blue solid line is mean performance. Contours indicate what fraction of classifiers in the evaluation category exceeded the shown performance. Gray area exceeds performance of all classifiers.

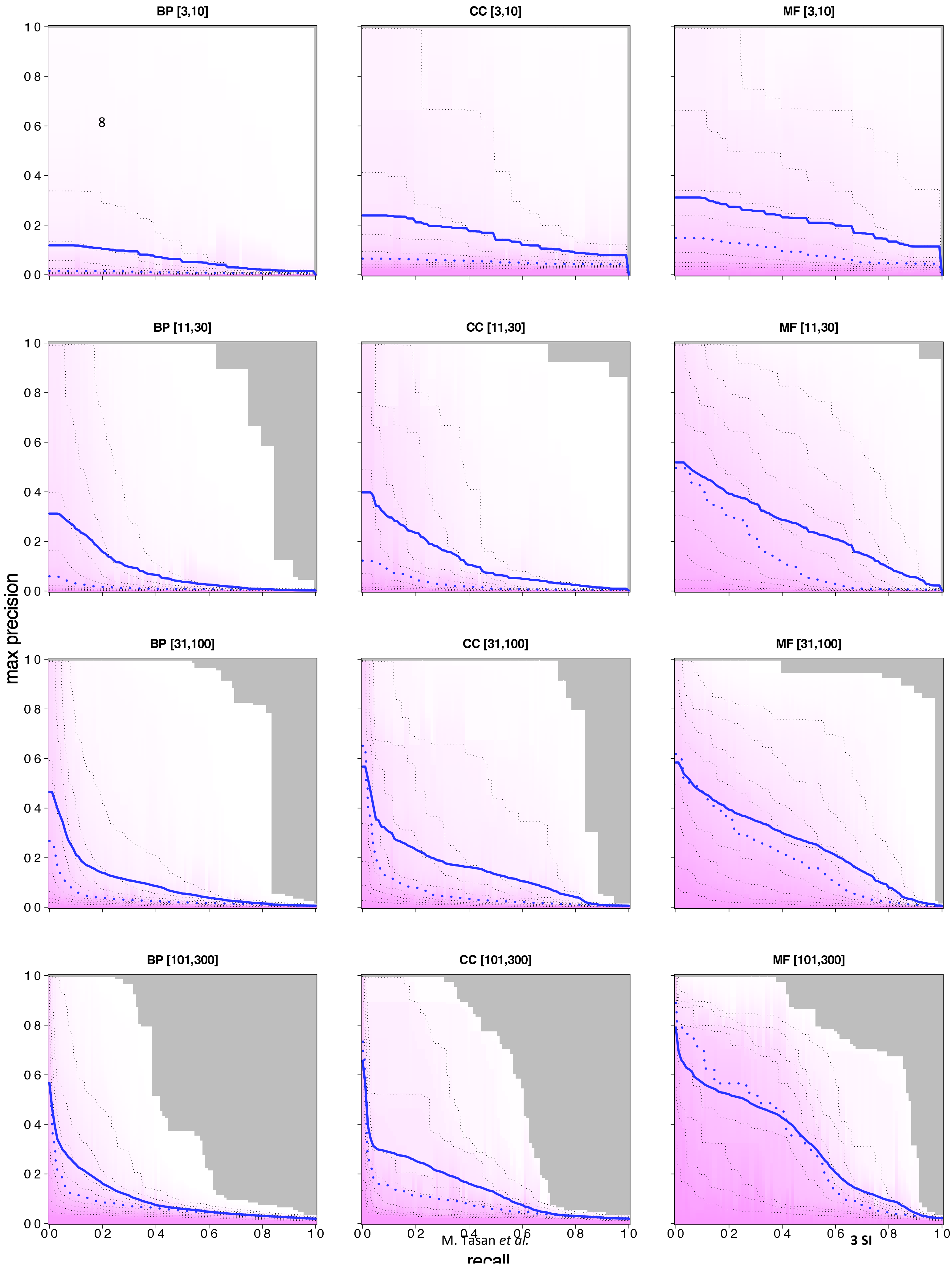
BP [3,10]
CC [3,10]
MF [3,10]
BP [11,30]
CC [11,30]
MF [11,30]
BP [31,100]
CC [31,100]
MF [31,100]
BP [101,300]
CC [101,300]
MF [101,300]
max precision
recall
8
0 0
0 2
0 4
0 6
0 8
1 0

Figure S2 Aggregated GBA performance for each of the twelve GO term categories. Dashed lines indicate each 10% contour, heavy dashed line is median (50% contour), heavy solid line is mean performance. Contours indicate what fraction of classifiers in the evaluation category exceeded the shown performance. Gray area exceeds performance of all classifiers.

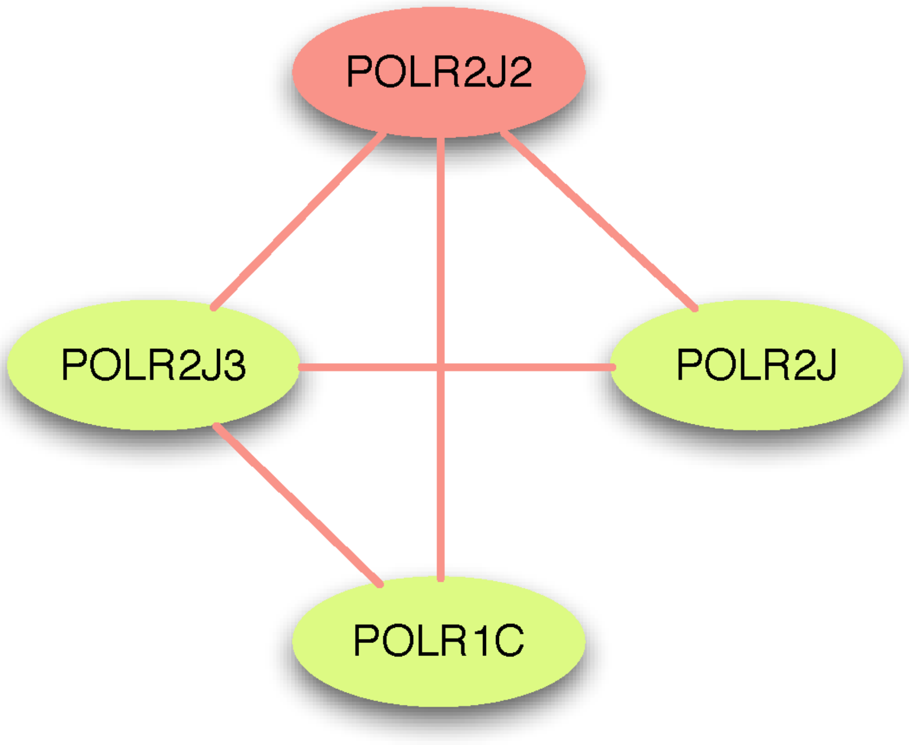

Figure S3 Network identified using 1 seed gene (in red) and the top 0.1% of edges from the BP [3, 30] FLN.

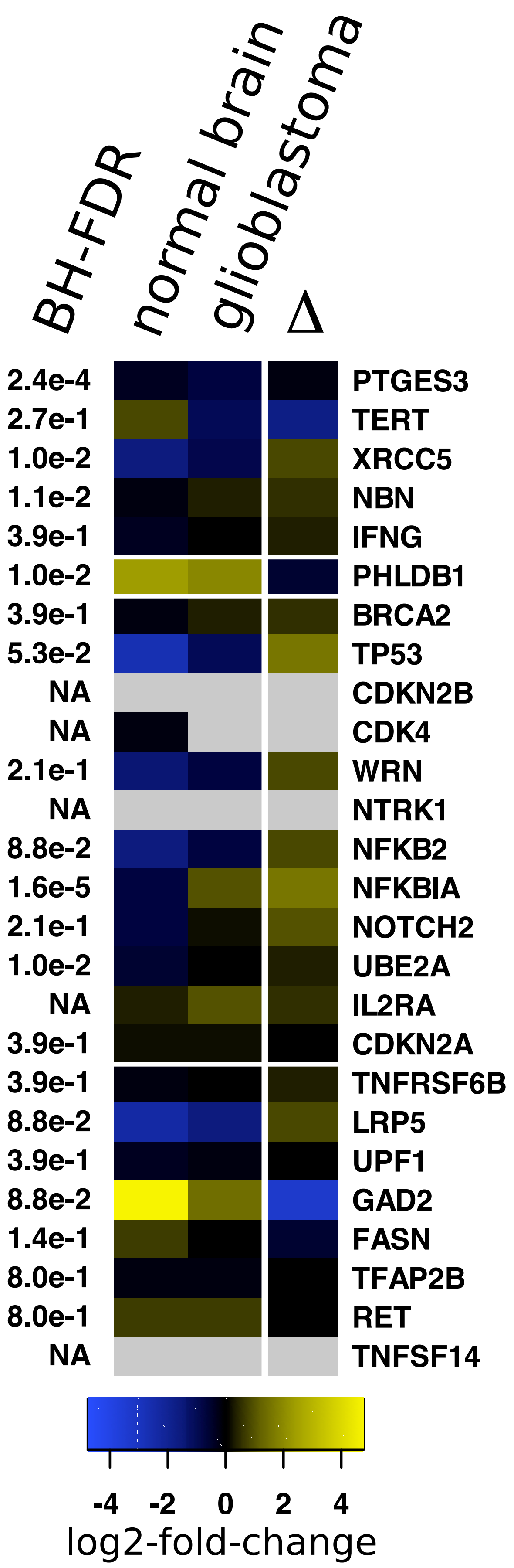
BH-FDR
normal brain
glioblastoma
Δ
2.4e-4 PTGES3
2.7e-1 TERT
1.0e-2 XRCC5
1.1e-2 NBN
3.9e-1 IFNG
1.0e-2 PHLDB1
3.9e-1 BRCA2
5.3e-2 TP53
NA CDKN2B
NA CDK4
2.1e-1 WRN
NA NTRK1
8.8e-2 NFKB2
1.6e-5 NFKBIA
2.1e-1 NOTCH2
1.0e-2 UBE2A
NA IL2RA
3.9e-1 CDKN2A
3.9e-1 TNFRSF6B
8.8e-2 LRP5
3.9e-1 UPF1
8.8e-2 GAD2
1.4e-1 FASN
8.0e-1 TFAP2B
8.0e-1 RET
NA TNFSF14
-4 -2 0 2 4
log2-fold-change

Figure S4 26 glioma network genes' mean microarray values in normal brain and glioblastomas (from NCBI's GEO dataset GDS1819 and ref. [8]). Test statistics computed using Welch's two-sample t-test with FDR adjustments for multiple testing.
