# Supplementary material for: A Resource of Quantitative Functional Annotation for Homo sapiens Genes
Source: G3 (Bethesda). 2012 Feb 1;2(2):223–33. doi: 10.1534/g3.111.000828 (PMC3284330; doi:10.1534/g3.111.000828)
Supplement: Supporting Information [file supp_2.2.223_FigureS2.pdf]

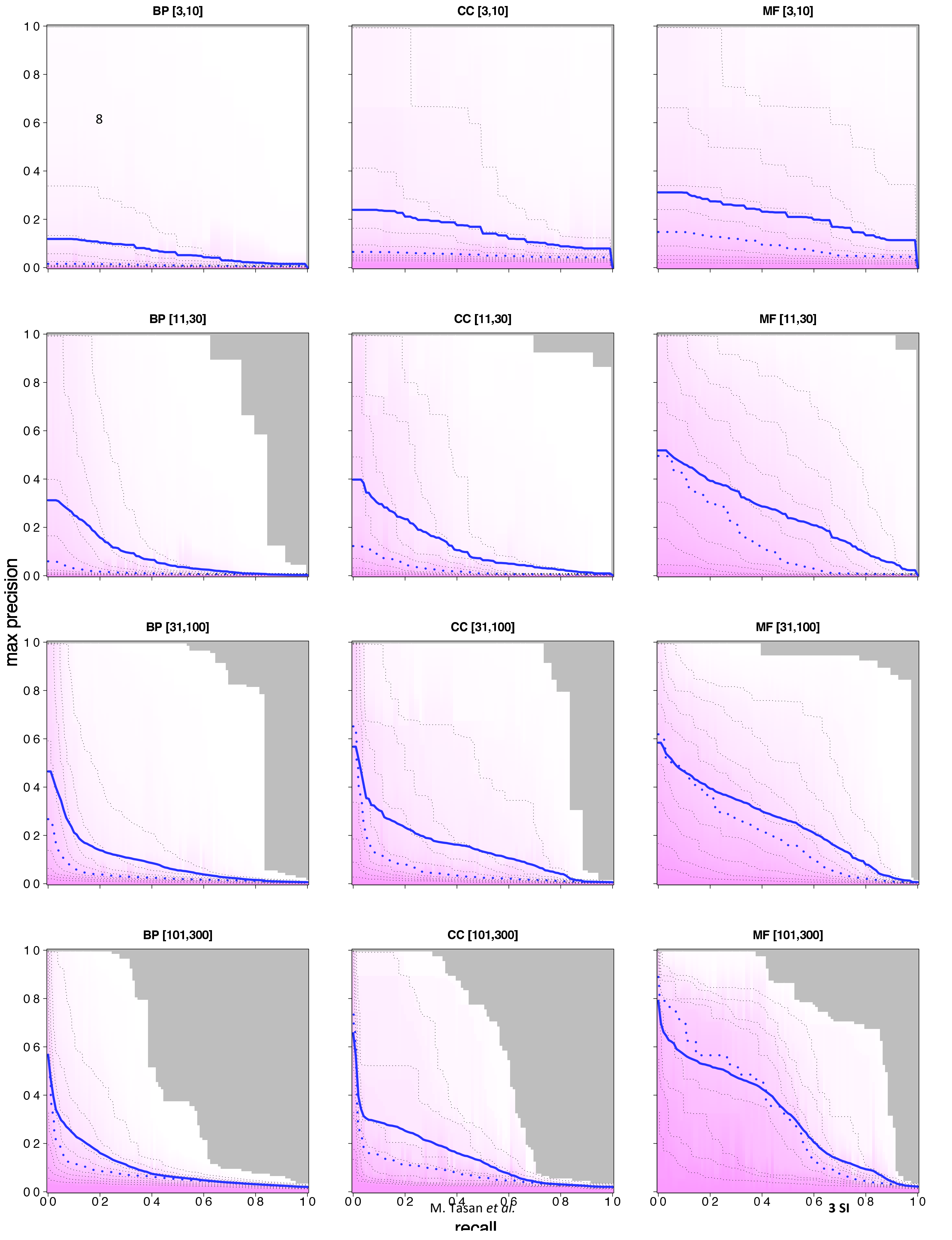
BP [3,10]
CC [3,10]
MF [3,10]
BP [11,30]
CC [11,30]
MF [11,30]
BP [31,100]
CC [31,100]
MF [31,100]
BP [101,300]
CC [101,300]
MF [101,300]
8
max precision
recall
0 0
0 2
0 4
0 6
0 8
1 0

Figure S2 Aggregated GBA performance for each of the twelve GO term categories. Dashed lines indicate each 10% contour, heavy dashed line is median (50% contour), heavy solid line is mean performance. Contours indicate what fraction of classifiers in the evaluation category exceeded the shown performance. Gray area exceeds performance of all classifiers.
