# Supplementary figures and images for: A Resource of Quantitative Functional Annotation for Homo sapiens Genes
Source: G3 (Bethesda). 2012 Feb 1;2(2):223–33. doi: 10.1534/g3.111.000828 (PMC3284330; doi:10.1534/g3.111.000828)

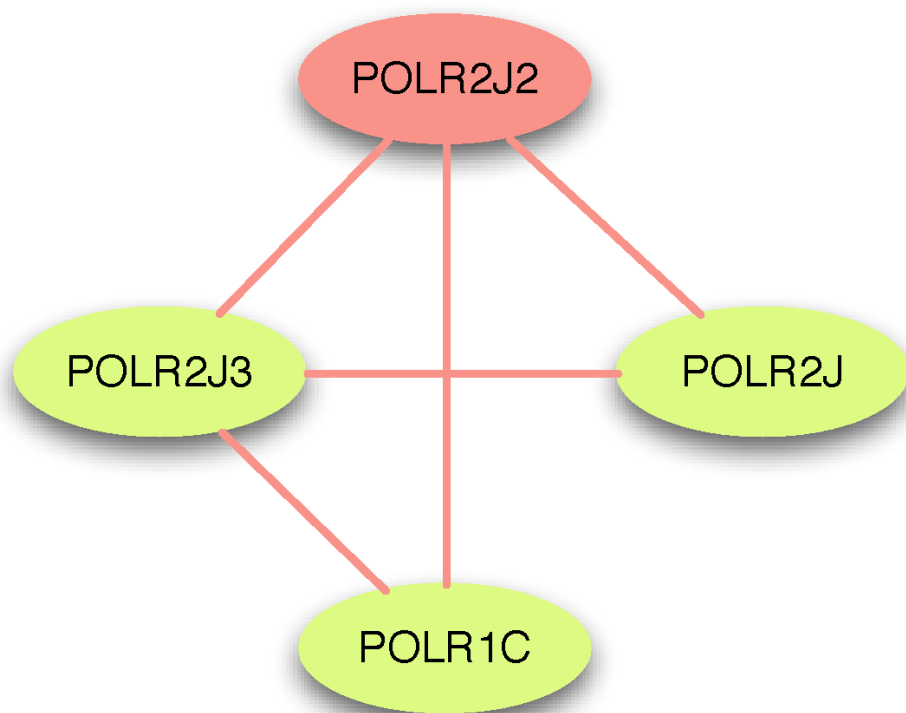

**Figure S3** Network identified using 1 seed gene (in red) and the top 0.1% of edges from the BP [3, 30] FLN.

Supplement: Supporting Information [file supp_2.2.223_FigureS3.pdf]
