# Supplementary material for: A Resource of Quantitative Functional Annotation for Homo sapiens Genes
Source: G3 (Bethesda). 2012 Feb 1;2(2):223–33. doi: 10.1534/g3.111.000828 (PMC3284330; doi:10.1534/g3.111.000828)
Supplement: Supporting Information [file supp_2.2.223_FigureS4.pdf]

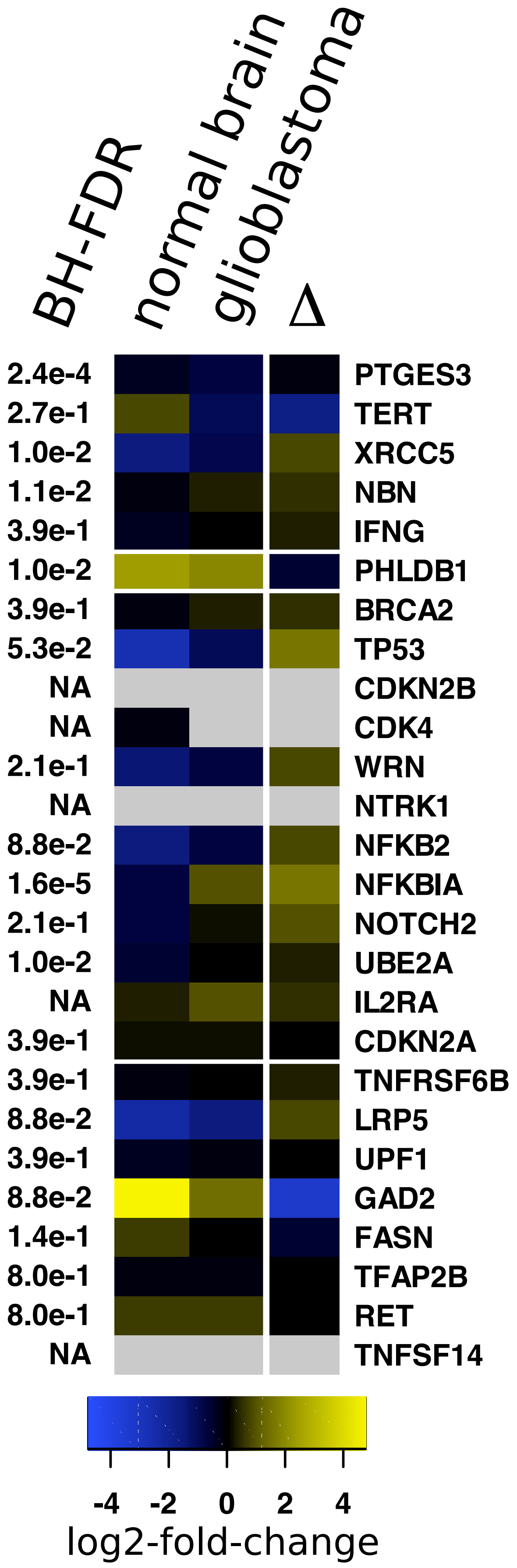
BH-FDR
normal brain
glioblastoma
Δ
2.4e-4 PTGES3
2.7e-1 TERT
1.0e-2 XRCC5
1.1e-2 NBN
3.9e-1 IFNG
1.0e-2 PHLDB1
3.9e-1 BRCA2
5.3e-2 TP53
NA CDKN2B
NA CDK4
2.1e-1 WRN
NA NTRK1
8.8e-2 NFKB2
1.6e-5 NFKBIA
2.1e-1 NOTCH2
1.0e-2 UBE2A
NA IL2RA
3.9e-1 CDKN2A
3.9e-1 TNFRSF6B
8.8e-2 LRP5
3.9e-1 UPF1
8.8e-2 GAD2
1.4e-1 FASN
8.0e-1 TFAP2B
8.0e-1 RET
NA TNFSF14
-4 -2 0 2 4
log2-fold-change

**Figure S4** 26 glioma network genes' mean microarray values in normal brain and glioblastomas (from NCBI's GEO dataset GDS1819 and ref. [8]). Test statistics computed using Welch's two-sample t-test with FDR adjustments for multiple testing.
